# Personality Traits, Clinical Characteristics, and Health-Related Quality of Life of Patients with Hypertension in a Primary Hospital in Ghana

**DOI:** 10.1155/2019/7489875

**Published:** 2019-01-02

**Authors:** Irene A. Kretchy, Franklin Acheampong, Jane Laryea, Joseph Osafo, Emmanuel Asampong, Erica Dickson

**Affiliations:** ^1^Department of Pharmacy Practice and Clinical Pharmacy, School of Pharmacy, College of Health Sciences, University of Ghana, Legon, Ghana; ^2^Department of Research, Korle Bu teaching Hospital, Accra, Ghana; ^3^Department of Psychology, School of Social Studies, College of Humanities, University of Ghana, Legon, Ghana; ^4^Department of Social and Behavioral Sciences, School of Public Health, College of Health Sciences, University of Ghana, Legon, Ghana

## Abstract

**Background:**

Hypertension is a major health problem that remains a significant threat to the health and general wellbeing of many people all over the world. In some patients, the etiology and prognosis of hypertension have been linked to psychological factors including personality traits. One primary goal of management is to improve the health-related quality of life (HRQoL) of patients with hypertension. This study aimed to examine the association between personality traits, clinical characteristics, and HRQoL in hypertension.

**Methods:**

A hospital-based cross-sectional quantitative study was conducted in a sample of 331 individuals with hypertension. Data on sociodemographic characteristics, clinical information, personality traits, and HRQoL were obtained from participants using an interviewer administered questionnaire.

**Results:**

The number of participants with a 1–10 years' duration of diagnosis for hypertension was highest (56.8%), with 52.9% having comorbidities such as diabetes (40.2%) and dyslipidaemia (20.9%). The average number of medications taken per patient was 2.14 (SD±0.79) and about 47.1% of the participants reported adequate medication adherence. Significant associations for age, education, monthly income, number of years with hypertension, and HRQoL were observed. While conscientiousness was significantly associated with all HRQoL domains, extraversion and agreeableness were significantly related to only the environmental domain.

**Conclusion:**

This study has demonstrated that clinical characteristics and patients' perception of their personality are relevant to their health-related quality of life outcomes. The findings suggest that when intervention efforts to improve the quality of life of patients with hypertension are being considered, a biopsychosocial approach should be employed. The implication is that treatment of hypertension in Ghana should be broadened to include the expertise of mental health professionals.

## 1. Background

Hypertension is a major health problem that remains a significant threat to the health and general wellbeing of many people all over the world. Even though there is a significant advancement in the detection, evaluation, and treatment of hypertension, the prevalence rates are high worldwide and in Ghana [1–4]. While hypertension is among the leading causes of hospital admissions, heart failure, renal failure, and death in Ghana, the primary goal of management is to reduce long term cardiovascular risk, reduce blood pressure, lessen risk of complications, and improve the quality of life of patients [5–7].

Quality of life is an important indicator when evaluating hypertensive treatment outcomes and health-related quality of life (HRQoL) is directly influenced by health-related issues which reflect the patient's burden associated with the disease [8–10]. Examples of such health-related issues include the coexistence of hypertension with other illnesses, total number of antihypertensive medications taken, extent of adherence to these medications, and the type of medications, although no class of antihypertensive agent offers a definite advantage over the others in terms of impact on HRQoL [11–15].

In some patients, hypertension has been reported to be a psychosomatic or behavioral disorder, where its etiology and prognosis have been linked to some psychological factors including personality traits [16–25]. Personality traits refer to relatively stable emotional, cognitive, and behavioral differences among individuals and have been shown to be highly consistent across adulthood [26]. The most common personality model explored has been the Big Five personality traits which suggests five broad domains used to describe human personality. The five dimensions comprising agreeableness, conscientiousness, extraversion, neuroticism, and openness to experience have shown various associations with health outcomes in patients with hypertension and cardiovascular diseases [10, 19, 27, 28].

While HRQoL may be compromised in patients with hypertension, there is limited body of research examining the determinants of HRQoL among persons with hypertension particularly in Ghana. This study aims to contribute to filling this gap by examining the association between personality, clinical characteristics, and HRQoL.

## 2. Methods

### 2.1. Study Design

A hospital-based cross-sectional study of patients with hypertension was carried out from December 2015 to January 2016.

### 2.2. Study Setting

The study was conducted at the Ledzokuku Krowor Municipal Assembly (LEKMA) Hospital in Teshie, an urban poor community in Accra, Ghana, which serves as a primary hospital for the people in the surrounding communities. The hospital runs specialized clinics on Tuesdays and Thursdays for patients with hypertension during which data were collected from the patients.

### 2.3. Study Participants

The minimum sample size was estimated at 312 using a projected prevalence of hypertension in an urban poor community in Ghana at 28.3% [3], a 95% confidence interval, and a standard value of 1.96 [29]. To compensate for losses, incomplete information, and low response rates from the participants, additional participants were enrolled for the study using a 10% nonresponse rate, but 19 questionnaires were rejected due to incomplete information. Therefore, information from a total number of 331 patients was used in data analysis.

Participants were eligible for inclusion if they were 18 years and older, were diagnosed with hypertension with/without comorbidities, and were on medications for at least 6 months prior to data collection.

### 2.4. Study Measures

The sociodemographic characteristics, clinical information, personality characteristics, medication adherence, and HRQoL information were obtained from participants using an interviewer administered questionnaire. A summary of the assessment areas and tools has been provided in Table 1. The questionnaire was translated from English to Ga and Twi which are the predominant local languages and were backtranslated into English by professional translators. Participants could choose the language of preference to respond to the questionnaire.

The sociodemographic characteristics included age, sex, marital status, education, employment status, and an estimated monthly income. Some medical information including period since diagnosed, presence of comorbidities, and number of medications taken were also obtained from the patient records.

The Ten Item Personality Inventory (TIPI) is a very brief measure of the Big Five personality domains and was used for the assessment of personality traits, comprising two items for each of the traits of agreeableness, conscientiousness, extraversion, neuroticism, and openness [30]. Each item was assessed using a 7-point response scale ranging from 1 = disagree strongly to 7 = agree strongly. The TIPI is scored using items 2R, 7 for agreeableness; 3, 8R for Conscientiousness; 1, 6R for Extraversion; 4R, 9 for Emotional Stability; and 5, 10R for Openness (‘R' represents the items that were reverse-scored). Each personality trait was classified as low (0.5–2.5), moderate (3.0–5.0) and high (5.5–7.0) based on the TIPI norms. The use of this personality instrument was guided by its potential to provide an easy assessment of personality in a clinical setting as compared to the existing ones which require longer duration to complete.

The Medication Adherence Questionnaire (MAQ) was used to assess medication adherence and the questions covered medication intake behavior of patients. The total score ranged from zero to four representing low (3-4), moderate (1-2), and high (0) adherence, respectively [31].

The HRQoL was measured using the shorter version of the WHO Quality of Life Scale (WHOQOL-BREF) which is a 26-item cross-cultural scale that covers the four domains of quality of life: psychological, physical, social relationships, and environmental domains [32]. The mean score of items within each domain was used to calculate the domain mean where higher scores denoted higher quality of life. The mean score of each domain was then multiplied by 4, making it comparable with the WHOQOL-100 [32]. The Cronbach alpha values of 0.81, 0.82, 0.68, and 0.80 have been reported for psychological health, physical health, social relationship, and environmental health, respectively [33].

To ensure the reliability and appropriateness of the questionnaire, a pilot study was conducted involving 30 participants. The TIPI was reliable with Cronbach alpha of 9.1, 9.2, 7.8, 7.8, and 8.8 for agreeableness, conscientiousness, extraversion, neuroticism, and openness, respectively. Similarly, the psychological (0.92), physical (0.88), social relations (0.90), and environmental (0.87) domains of HRQoL scale as well as the MAQ (0.75) were reliable.

### 2.5. Data Analysis

The data were entered into SPSS version 20 for analysis. Descriptive analysis was carried out to determine frequencies, means, and standard deviations. Linear regression analysis and a three-step hierarchical regression model were performed to evaluate the effect of clinical characteristics on HRQOL dimensions [10]. During the first step, demographic characteristics (age, sex, monthly income, employment status, and education) were entered. In step two, comorbidity, number of medications, and number of years with hypertension were added to the demographics and in the final step medication adherence was included in the model. Finally, univariate analyses were performed among personality traits, adherence, and HRQoL dimensions.

## 3. Results

### 3.1. Characteristics of Patients

Table 2 presents the frequency and percentages of patient characteristics in the study. The majority of the participants were females (81.9), were above 59 years of age (55%), were married (52%), had attained up to the secondary school level of education (62.2%), were unemployed (58.6%), and make an average monthly income of less than 25 US Dollars (64%).

The number of participants with a 1–10 years' duration of diagnosis for hypertension was highest (56.8%), followed by 11–20 years (20.2%), and least for >40 years (1.8%). The presence of comorbidities was high (52.9%) with the following comorbidities recorded: diabetes (40.2%), dyslipidaemia (20.9%), stroke (10.7%), peptic ulcer (7.4%), osteoarthritis (4.1%), ischaemic heart disease (3.7%), asthma (3.3%), urinary tract infection (2.9%), anaemia (2.1%), gastritis (2.1%), gout (0.8%), lumbago (0.4%), benign prostatic hyperplasia (0.4%), and goitre (0.4%).

The number of medications taken per patient was noted (mean = 2.14 ± 0.79). The average number of antihypertensive medications taken per patient was mean = 2.89 ± 1.09 with the following frequencies: Amlodipine (n = 219), Lisinopril (n = 212), Nifedipine (n = 201), Bendrofluazide (n = 184), Atenolol (n = 42), Losartan (n = 14), propranolol (n = 12), Frusemide (n = 8), carvedilol (n = 8), Candesartan (n = 6), Methyldopa (n = 5), and Hydrochlorothiazide (n = 4). Patients who adequately adhered to their medications were 47.1%.

### 3.2. Patient Characteristics and Health-Related Quality of Life

Table 3 shows the linear regression analysis between the study variables and the different domains of HRQoL with significant associations for age (p < 0.05), education (p < 0.001), and the physical domain. For the psychological domain, the model showed significant relationships with education (p < 0.05) and monthly income (p < 0.001). Age (p < 0.001) and education (p < 0.05) were significantly associated with the social and environmental domains, respectively. There was no statistically significant relationship between adherence and quality of life (p > 0.05).

Table 4 shows the statistically significant results of the linear regression analysis of each of the quality of life dimensions and demographics, clinical factors, and adherence. The demographic variables did not explain much of the variance of HRQoL with the largest effect seen on the physical dimension.

The result of the Pearson product-moment correlation analysis showed that conscientiousness was significantly associated with all the dimensions of HRQoL. Extraversion and agreeableness were also significantly associated with the environmental dimension of HRQoL (Table 5).

Only the conscientiousness personality trait correlated statistically significantly with adherence to medication (Table 6).

## 4. Discussions

The present study examined the association between clinical characteristics, personality traits, and health-related quality of life outcomes. While 47.1% of patients adequately adhered to their medications, the findings revealed that age, education, monthly income, and presence of comorbid health conditions were associated with HRQoL. In relation to the Big Five personality characteristics, agreeableness, conscientiousness, and extraversion were also associated with HRQoL while only conscientiousness was related significantly to medication adherence.

Some important observations on the demographics showed that most women reporting to the hospital were above the age of 50 years and had the lowest level of income. A plausible reason for this observation could be the likelihood of health seeking behavior among women although they may be in the lower socioeconomic level [34, 35].

An inverse relationship between age and HRQoL was found in this study and this is consistent with findings from previous studies [19, 36]. This may be because hypertension is more common in the elderly [37] and as age increases the period of having hypertension is also prolonged resulting in a build-up of factors that could negatively impact on particularly the physical aspect of quality of life of a patient over a period. Similarly, some studies have identified hypertension as a risk factor for a decline in the quality of life of the elderly population [38–40].

The association between educational level and quality of life has been reported [41, 42], where like the current study education was associated with HRQoL outcomes, particularly for the physical and environmental domains. This observation may be explained by the fact that patients who are educated tend to be knowledgeable about their conditions, leading to lower levels of anxiety, increased physical functioning, and better general health ratings which improve HRQoL [43].

Our results indicated that monthly income was negatively associated with the physical domain of HRQoL. Income and low socioeconomic status have been associated with poor quality of life, and undesirable health outcomes such as poor general health, decline in mobility, social isolation, poor emotional coping, and long-term disability [44, 45].

Coexisting diseases may have considerable effects on patients' well-being. Individuals with chronic conditions usually have comorbidities [46]. The findings of this study indicated that the majority of participants had comorbidities which had a significant relationship with their HRQoL especially in the physical domain. The inverse relationship between comorbidity and quality of life has also been reported [7, 47].

Personality has been proposed to be relatively stable emotional, cognitive, and behavioral characteristics of an individual which is consistent across adulthood [26]. Since personality is a relatively stable inclination of a person to interpret different circumstances, it is probable that the personality of patients with hypertension play a role in determining their HRQoL. Our findings add to the literature on the possible association between personality and HRQoL by showing that conscientiousness relates significantly with all domains of HRQoL, while agreeableness and extraversion were associated with the environmental domain. Conversely, neuroticism, which refers to the propensity of an individual to experience negative affect, and openness to experience, which also refers to creativity and originality, did not predict HRQoL in this group of patients.

The association between conscientiousness and quality of life has been demonstrated for all and some domains of quality of life [18, 48] with some contradicting views [10, 19]. It is remarkable to note that conscientious individuals are more devoted to maintaining their roles, having subtraits of self-efficacy, order, dutifulness, and self-discipline [49]. It also describes the extent to which one works towards goals in a disciplined manner and this may have also contributed to the association between conscientiousness and medication adherence observed in this study. Our findings are important because it concerns patients with hypertension who as part of their goals of management have to adhere to both pharmacological and lifestyle behaviors to control blood pressure, reduce risk of complications, and improve quality of life [6]. The more orderly and organized the patients are, the more critical it is for the performance of habits and routines for better quality of life outcomes.

Agreeableness, which refers to the degree of selflessness or hostility towards others, and extraversion, which is characterized by attributes such as sociability, also showed significant associations with the environmental domain of HRQoL. The observed positive association implied that high scores on agreeableness and extraversion were related to better environmental quality of life. Considering the fact that agreeableness could be useful for harmony in many environments [50], it suggests that the patients may be cooperating and considerate towards the environmental factors in relation to their health provision, leading to desirable HRQoL outcomes [51]. Similarly, extraverts enjoy interacting with people and are often perceived as being full of life, energy, and positivity and these attributes may impact on quality of life. The associations between agreeableness, extraversion, and quality of life have also been reported elsewhere [10, 52].

This study acknowledges the limitation that it employed a quantitative approach and though an adequate sample was used, some in-depth interviews could have been conducted to really understand the personality traits in illness representations, clinical parameters, and the dimensions of quality of life. Thus, future studies could use a mixed method approach to understand the relationship between clinical characteristics, personality traits, and quality of life.

### 4.1. Conclusion

This study has demonstrated that clinical characteristics and patients' perception of their personality are relevant to their health-related quality of life outcomes. The findings suggest that when intervention efforts to improve the quality of life of patients with hypertension are being considered, a biopsychosocial approach should be employed. The implication is that treatment of hypertension in Ghana should be broadened to include the expertise of mental health professionals such as clinical and health psychologists.

## Figures and Tables

**Table 1:** Assessment areas and tools.

Assessment area	Assessment tool
Sociodemographics	Age, sex, marital status, education, employment status, and estimated monthly income
Clinical factors	Period since diagnosed, presence of comorbidities, and number of medications taken
	Medication adherence using Medication Adherence Questionnaire
Personality trait	Ten Item Personality Inventory
Health-related quality of life	WHO Quality of Life (WHOQOL-BREF) scale

**Table 2:** Characteristics of patients (n=331).

Characteristics	Number	Percentage
Gender		
Male	60	18.1
Female	271	81.9
Age Ranges (years)		
	2	0.6
20 – 29	2	0.6
30 - 39	10	3.0
40 - 49	45	13.6
50 – 59	90	27.2
	182	55.0
Marital status		
Single	16	4.8
Married	172	52.0
Widowed	100	30.2
Divorced	43	13.0
Level of education		
None	84	25.4
Primary	21	6.4
Secondary	206	62.2
Postsecondary	15	4.5
Tertiary	5	1.5
Employment status		
Unemployed	194	58.6
Employed	115	34.7
Retired	22	6.7
Monthly income ($)		
	212	64.0
26-125	78	23.6
126-250	37	11.2
251-500	3	0.9
	1	0.3
Period since diagnosed (years)		
	49	14.8
1–10	188	56.8
11–20	67	20.2
21–40	21	6.3
	6	1.8
Presence of Comorbidity		
Yes	175	52.9
No	156	47.1
Medication adherence		
High	156	47.1
Poor	175	52.9

Number of medications taken per patient, Mean (±S.D) = 2.14 (0.79)

**Table 3:** Adjusted mean differences (and 95% confidence intervals) from a linear regression analyses evaluating the effect of sociodemographic/economic factors, presence of comorbidity, number of years with hypertension, and number of medications taken on HRQOL dimensions.

	**Physical **	**Psychological **	**Social **	**Environmental **
**Gender **				
Male	*Ref*			
Female	1.75 (-1.36, 4.88)	2.25 (1.77, 6.28)	0.52 (-1.41, 2.44)	3.32 (-1.43, 8.06)
**Age**	*∗*		*∗*	
	*Ref*			
20 – 29	-3.94 (-23.77, 15.88)	4.57 (-20.99, 30.13)	13.19 (-25.40, -0.97)	-14.62 (-44.72, 15.49)
30 - 39	-7.7 (-22.96, 7.55)	6.01 (-13.66, 25.68)	13.96 (-23.36, -4.56)	-10.91 (-34.07, 12.25)
40 - 49	3.07 (-11.49, 17.65)	12.38 (-6.41, 31.16)	8.27 (-17.25, 0.70)	-5.56 (-27.68, 12.25)
50 – 59	-2.89 (-17.24, 11.45)	11.40 (-7.09, 29.89)	9.89 (-18.72, -1.05)	-7.06 (-28.84, 14.71)
	-1.56 (-15.90, 12.78)	13.00 (-5.49, 31.49)	9.54 (-18.38, -0.71)	-8.40 (-30.17, 13.37)
**Education**	*∗∗∗*	*∗*		*∗*
None	*Ref*			
Primary	1.9 (-2.90, 6.71)	-1.31 (-7.51, 4.89)	2.26 (-0.70, 5.22)	6.33 (-0.97, 13.63)
Secondary	6.12 (3.40, 8.83)	2.59 (-0.91, 6.08)	2.20 (0.53, 3.87)	6.35 (2.23, 10.46)
Post-secondary	7.15 (2.05, 12.26)	6.42 (-0.15, 13.00)	1.09 (-2.06, 4.23)	9.64 (1.90, 17.39)
Tertiary	7.27 (1.78, 12.76)	3.08 (-10.16, 4.00)	0.86 (-2.53, 4.24)	5.56 (-2.78, 13. 90)
**Monthly income**		*∗∗∗*		
None/Pension	*Ref*			
	2.1 (-2.17, 6.38)	2.42 (-7.93, 3.10)	2.26 (-4.90, 0.38)	-2.08 (-8.58, 4.41)
500-999	0.86 (-3.12, 4.85)	0.47 (-4.68, 5.60)	0.29 (-2.17, 2.75)	3.76 (-2.30, 4.42)
1000 +	-2.59 (-9.95, 4.77)	17.55 (8.06, 27.04)	2.25 ( -2.28, 6.79)	5.37 (-5.80, 16.55)
**Employment **				
Unemployed	*Ref*			
Employed	0.91 (-2.64, 4.47)	-0.50 (-5.09, 4.09)	0.90 (-1.29, 3.09)	-0.33 (-5.74, 5.07)
Retired	2.04 (-3.34, 7.42)	2.52 (-4.42, 9.47)	1.43 (-1.89, 4.75)	7.09 (-1.09, 15.26)
**Comorbidity**				
No	*Ref*			
Yes	-1.7 (-4.00, 0.59)	0.39 (-2.57, 3.35)	-0.48 (-1.89, 0.04)	-0.25 (-3.72, 3.25)
**Years with hypertension**		*∗*		
	*Ref*			
1-9 years	0.77 (-2.43, 3.98)	5.97 (1.83, 10.10)	1.01 (-0.96, 2.99)	-0.25 (-5.12, 4.62)
10-19 years	0.66 (-3.27, 4.61)	2.23 (-2.86, 7.32)	-0.27 (-2.71, 2.16)	-2.40 (-8.39, 3.59)
20+ years	0.19 (-4.87, 5.27)	2.67 (3.87, 9.21)	-0.89 (-4.01, 2.24)	4.49 (-3.20, 12.20)
**Adherence **				
Poor	*Ref*			
High	-0.073 (-0.052, 0.183)	0.009 (0.002, 0.875)	-0.011 (-0.007, 0.842)	-0.073 (-0.009, 0.185)

^*∗*^p<0.05, ^*∗∗*^p<0.01, and ^*∗∗∗*^p<0.001.

**Table 4:** Hierarchical regression model of demographic characteristics, clinical variables, and HRQoL domains.

	Physical	Psychological	Social	Environmental
Step 1 R^2^	5.2%	0.8%	0.7%	3.2%
Step 2 R^2^ (R^2^ change)	6.1% (0.9%)	1.0% (0.2%)	1.3% (0.6%)	3.2% (0%)
Step 3 R^2^ (R^2^ change)	6.5% (0.4%)	1.0% (0.0%)	1.3% (0.0%)	3.6% (0.4%)
Age	-0.201(0.028)	0.146 (0.115)	- 0.13 (0.235)	- 0.040(0.132)
Sex	0.114 (0.365)	0.013 (0.471)	- 0.042 (0.479)	0.022(0.419)
Education	0.152(0.003)	0.024 (0.258)	0.016(0.103)	0.124(0.010)
Employment status	-0.075(0.086)	0.013 (0.407)	-0.032(0.282)	0.016(0.383)
Monthly income	-0.128(0.001)	0.022 (0.365)	-0.003(0.447)	-0.040(0.050)
Co-morbidity	0.102 (0.032)	- 0.046 (0.200)	0.071 (0.100)	0.013(0.409)
Number of medications taken	-0.006 (0.455)	0.023 (0.452)	0.024 (0.303)	- 0.074 (0.090)
Number of years with hypertension	-0.120 (0.138)	0.023 (0.335)	- 0.039 (0.090)	- 0.023(0.462)
Adherence	-0.254 (0.270)	0.010 (0.298)	-0.025 (0.859)	-0.409 (0.232)

Step 1: only demographics (Age, sex, monthly income, employment status, and education) were entered; step 2: Comorbidity, number of medications, and number of years with hypertension were added to the demographics; step 3: adherence was then added to the other variables.

**Table 5:** Univariate analysis of the Big Five Personality Traits and HRQoL domains.

	Physical	Social	Environmental	Psychological
Extraversion	0.08(0.17)	0.11(0.05)	0.21 (<0.001)	0.10(0.083)
Agreeableness	0.11(0.06)	0.06(0.25)	0.11(0.05)	-0.004(0.95)
Conscientiousness	0.12(0.03)	0.16(0.003)	0.26(<0.001)	0.17(0.002)
Emotional stability	0.05(0.34)	0.08(0.16)	0.08(0.14)	0.04(0.46)
Openness to experience	0.04(0.51)	-0.09(0.12)	0.08(0.18)	0.01(0.80)

Correlation coefficients (p value).

**Table 6:** Univariate analysis between the Big Five Personality traits and level of adherence.

	Correlation Coefficients	p value
Extraversion	-0.062	0.259
Agreeableness	0.002	0.970
Conscientiousness	0.118	0.032
Emotional stability	-0.075	0.175
Openness to experience	-0.105	0.057

p value<0.05.

## Data Availability

The datasets during and/or analysed during the current study are available from the corresponding author on reasonable request.
